# Skilled antenatal care services utilisation in sub-Saharan Africa: a pooled analysis of demographic and health surveys from 32 countries

**DOI:** 10.1186/s12884-022-05137-5

**Published:** 2022-11-10

**Authors:** Kwamena Sekyi Dickson, Joshua Okyere, Bright Opoku Ahinkorah, Abdul-Aziz Seidu, Tarif Salihu, Vincent Bediako, Bernard Afriyie Owusu, Eugene Budu, Wonder Agbemavi, Jane Odurowaah Edjah, Eugene Kofuor Maafo Darteh

**Affiliations:** 1 Department of Population and Health, University of Cape Coast, Cape Coast, Ghana; 2 Department of Nursing, College of Health Sciences, Kwame Nkrumah University of Science and Technology, Kumasi, Ghana; 3 School of Public Health, Faculty of Health, University of Technology Sydney, Ultimo, Australia; 4 Centre for Gender and Advocacy, Takoradi Technical University, Takoradi, Ghana; 5 College of Public Health, Medical and Veterinary Sciences, James Cook University, Townsville, Australia; 6 Ghana Health Service, Ewim Polyclinic, Central Region, Cape Coast, Ghana; 7 Korle Bu Teaching Hospital, P. O. Box, 77, Accra, Ghana; 8 Guidance and Counselling Centre, University of Cape Coast, Cape Coast, Ghana

**Keywords:** Skilled, Antenatal care, Sub-Saharan Africa, Public health, Social demography

## Abstract

**Background:**

Each day, an estimated 800 women die from preventable pregnancy and childbirth related complications, where 99% of these avoidable deaths happen in low-and middle-income countries. Skilled attendance during antenatal care (ANC) plays a role in reducing maternal and child mortality. However, the factors that predict the utilisation of skilled ANC services in sub-Saharan Africa (SSA) remains sparsely investigated. Therefore, we examined women’s utilisation of skilled ANC services in SSA.

**Methods:**

The research used pooled data from the most recent Demographic and Health Surveys conducted in 32 countries in SSA between January 1, 2010, and December 31, 2019. Binary logistic regression was used to examine the predictors of skilled ANC services utilisation. The results are presented as crude and adjusted odds ratios (aOR) with 95% confidence interval (CI).

**Results:**

The prevalence of skilled ANC services utilisation in SSA was 76.0%, with the highest and lowest prevalence in Gambia (99.2%) and Burundi (8.4%), respectively. Lower odds of ANC from skilled providers was found among women aged 45–49 compared to those aged 20–24 (aOR = 0.86, CI = 0.79–0.94); widowed women compared to married women (aOR = 0.84, CI = 0.72–0.99); women who consider getting permission to visit the health facility as a big problem compared to those who consider that as not a big problem (aOR = 0.74, CI = 0.71–0.77); women who consider getting money needed for treatment as not a big problem compared to those who consider that as a big problem (aOR = 0.84, CI = 0.72–0.99); and women who consider distance to the health facility as a big problem compared to those who consider that as not a big problem (aOR = 0.75, CI = 0.72–0.77).

**Conclusion:**

SSA has relatively high prevalence of skilled ANC services utilisation, however, there are substantial country-level disparities that need to be prioritised. Increasing maternal reproductive age being widowed and far distance to health facility were factors that predicted lower likelihood of skilled ANC services utilisation. There is, therefore, the need to intensify female formal education, invest in community-based healthcare facilities in rural areas and leverage on the media in advocating for skilled ANC services utilisation.

## Background

Globally, there has been significant improvement in maternal health outcomes. This is evident in the 45% decline in the global maternal mortality ratio (MMR) from 380 to 210 per 100,000 between 1990 and 2013 [1]. Sub-Saharan Africa (SSA) has also achieved nearly 40% decline in MMR since 2000 [2]. Albeit this milestone recorded, the average MMR worldwide is far above the targets of the Sustainable Development Goal (SDG) 3.1 which seeks to reduce MMR to 70 per 100,000 live births. Additionally, there are significant disparities in MMR across the globe. While the high-income countries reported a MMR of 11 per 100,000 live births in 2017; low income countries reported MMR of 462 per 100,000 live births in the same year [2]. SSA alone accounts for nearly 66% of the global MMR [3]. This development calls for more commitment to accelerate efforts to reduce MMR in SSA. Evidence shows that three-quarters (75%) of all maternal death are due to pregnancy related complication which could have been averted if women utilised appropriate maternal health services throughout the continuum of care [4].

Antenatal care (ANC) is one of those maternal healthcare services that reduces the risk of maternal mortality. ANC is a sort of preventive care that aims to provide frequent check-ups that allow physicians or midwives to manage and prevent any health concerns during pregnancy while also promoting healthy lifestyles that benefit both mother and child [5]. During ANC sessions, women receive health education on the need to practice exclusive breastfeeding, as well as how they can prevent pregnancy related complications [6]. The WHO recommends that ANC be initiated within the first trimester of pregnancy with at least four visits, and optimally eight visits [7]. Moreover, ANC services are expected to be delivered by skilled healthcare providers [8].

When pregnant women seek ANC services from skilled healthcare providers (i.e., doctor, nurse, nurse/midwife, auxiliary midwife, community health nurse/officer), it is termed as skilled ANC utilisation [5]. Evidence available indicates that about 90% of women worldwide utilise skilled ANC services at least once; however, only 60% of women utilise skilled ANC services for at least four times, which is the WHO recommended minimum ANC visit [9]. The statistics are more staggering in SSA where only 49% of women utilise at least four times skilled ANC services [9]. This underscores the need to investigate and understand the nuances that exist in the utilisation of skilled ANC services in SSA.

Previous studies that have attempted to examine the skilled ANC utilisation have done so from a country-specific basis [5, 6, 8]. As such, the dynamics from a regional perspective is missing. For instance, Dickson et al. [5] investigated women’s utilisation of skilled ANC services in Ghana which revealed that 88% of women utilised skilled ANC. Their study showed that maternal education, wealth status, place of residence, parity and ethnicity determined the utilisation of skilled ANC. Other studies conducted within SSA examined the determinants of clients’ satisfaction to skilled ANC services [10], and male partner attendance of skilled ANC services [11]. To the best of our knowledge, there are no other studies within the SSA region that has investigated women’s utilisation of skilled ANC services. Thus, creating significant gap in knowledge. We, therefore, pose two main questions: (a) what proportion of women in SSA utilise skilled ANC services? (b) what factors predict women’s utilisation of skilled ANC services? The aim of this study is to examine women’s utilisation of skilled ANC services in SSA using the most recent nationally representative surveys of 32 countries.

## Methods

### Data source and study design

The research used pooled data from the most recent Demographic and Health Surveys (DHS) conducted in 32 countries in SSA between January 1, 2010, and December 31, 2019. The DHS is a countrywide representative study conducted in many LMICs usually over a five-year period. The DHS focuses on issues related to maternal and child health. In areas such as sampling, questionnaires, data collection, cleaning, coding, and analysis, the DHS follows standardized procedures which allow for comparability across countries. The survey utilizes a two-stage cluster sampling technique with the detailed sampling process highlighted in a previous study [12]. For this study, 190,903 women who had given birth in the five years prior to the surveys were included. In the case of multiple births in the last five years, the last pregnancy that led the birth was included. The dataset is freely available for download at https://dhsprogram.com/data/available-datasets.cfm. We relied on the “Strengthening the Reporting of Observational Studies in Epidemiology” (STROBE) guideline in writing the manuscript [13].

### Definition of variables

#### Outcome variable

The study used skilled ANC services utilisation as the outcome variable. The skilled ANC services utilisation variable was derived from the response to the question “did you see anyone for antenatal care for this pregnancy? If YES: whom did you see?” Responses were categorised under Health Personnel and Other Person. Health Personnel included Doctor, Nurse, Nurse/Midwife, Auxiliary Midwife, Community Health Nurse/Officer. Other persons also consisted of Traditional Birth Attendant (TBA), Traditional Health Volunteer, Village Health Volunteer, Other. For the purpose of the study, skilled ANC services utilisation was defined as utilising the services of a Doctor, Nurse, Auxiliary Midwife, Nurse/Midwife during antenatal care [5, 14].

#### Explanatory variables

The study used eleven explanatory variables, these were maternal age, place of residence, level of education, wealth status, marital status, partners’ education, frequency of listening to radio and watching television, getting medical help for self: getting permission to visit the health facility, getting money needed for treatment and distance to health facility. These variables were used in accordance with both theoretical and empirical literature [5, 10, 11, 14]. For instance, one study has showed that higher frequency of listening to radio or watching TV was associated with higher utilisation of skilled ANC services as the individual is exposed to health information through these media sources [5]. Age was classified into 15 – 19, 20 – 24, 25 – 29, 20 – 34, 35 – 39, 40 – 44, 45 – 49. Type of residence was coded as Urban or Rural. Educational level and partners educational level were classified into no education, primary, secondary, and higher education. Marital status was captured as never married, married, living with a partner, widowed, divorced, and separated. Wealth status was grouped into poorest, poorer, middle, richer, and richest. Frequency of listening to radio and watching television were captured as not at all, less than once a week, at least once a week, and almost every day. Getting medical help for self: getting permission to visit the health facility, getting money needed for treatment, and distance to health facility were categorised as a big problem or not a big problem.

### Data analyses

Frequencies and percentages were used to show the prevalence of skilled ANC services utilisation and its distribution across the explanatory variables. To illustrate how the explanatory variables are associated with skilled antenatal care, a discrete choice model (binary logistic regression) was used. Binary logistic regression was used precisely because the model is ideally suited to dichotomous variables and its ability to forecast a combination of continuous and categorical variables [5]. To cater for the between country variations, we controlled for survey country by including it in the logistic regression models. Results were presented as crude odds ratio (cOR) and adjusted odds ratio (aOR) at 95% confidence interval (CI) for Model 1 and Model 2, respectively. Inherent sampling weight was applied to deal with under-and over-sampling. All sub sections of explanatory variables with the highest frequency were used as the reference categories.

## Results

### Proportion of women who received skilled antenatal care services in sub-Saharan Africa

The prevalence of skilled ANC services utilisation in sub-Saharan Africa was 76.0%, with the highest and lowest prevalence in Gambia (99.2%) and Burundi (8.4%), respectively (Table 1).

**Table 1:** Proportion of women who receive skilled antenatal care in sub-Saharan Africa

Country	Frequency	Percentage	Proportion of women who utilized the services of skilled personnel during antenatal care
1. Angola 2015 – 2016	5,674	3.0	81.1
2. Benin, 2017 – 2018	7,957	4.2	82.5
3. Burundi, 2016 – 2017	7,856	4.1	8.4
4. Burkina Faso 2010	10,074	5.4	33.4
5. Cameroon, 2018	5,152	2.7	82.3
6. Chad, 2014 – 2015	3,398	1.8	56.1
7. Comoros, 2012	1,911	1.0	92.4
8. Congo DR, 2013 – 2014	10,092	5.3	88.6
9. Congo 2011 – 2012	5,034	2.6	86.2
10. Cote d’lvoire, 2011 – 2014	4,343	2.2	90.3
11. Ethiopia, 2016	7,070	3.7	48.1
12. Gabon, 2012	2,545	1.3	95.3
13. Ghana, 2014	3,682	1.9	90.3
14. Gambia, 2013	4,893	2.5	99.2
15. Guinea, 2018	5,030	2.6	80.2
16. Kenya, 2014	6,196	3.3	98.8
17. Lesotho, 2014 – 2015	2,251	1.2	95.5
18. Liberia, 2013	3,832	2.0	95.7
19. Malawi, 2015 – 2016	11,050	5.8	95.0
20. Mali, 2018	6,130	3.2	79.0
21. Mozambique, 2011	6,988	3.7	90.3
22. Namibia, 2013	1,830	1.0	96.4
23. Nigeria, 2018	20,320	10.7	66.8
24. Niger, 2012	7,821	4.1	82.9
25. Rwanda, 2014 – 2015	5,381	2.8	99.2
26. Sierra Leone, 2019	5,814	3.1	97.9
27. South Africa, 2016	616	0.3	11.0
28. Togo, 2013 – 2014	4,525	2.4	49.6
29. Tanzania, 2016	5,684	3.0	11.0
30. Uganda, 2016	8,048	4.2	97.5
31. Zambia, 2018	5,313	2.9	97.1
32. Zimbabwe, 2015	4,166	2.2	93.2
**All Countries**	**190,903**	**100**	**76.0**

### Distribution of skilled antenatal care services utilisation across background characteristics of respondents

In terms of the distribution of skilled ANC services utilisation across background characteristics of respondents, the highest prevalence of skilled ANC services utilisation was observed among women aged 20–24 (77.2%), those in urban areas (90.0%), women with higher education (97.3%), those with richest wealth quintile (89.3%), separated women (89.4%) and women whose partners had higher education (94.4%). We also found the highest prevalence of skilled ANC provision among women who listened to radio almost every day (86.5%), those who watched television almost every day (95.7%), women who considered getting permission to visit the health facility as not a big problem (77.4%), those who considered getting money needed for treatment as not a big problem (81.7%) and women who considered distance to the health facility as not a big problem (79.6%) (see Table 2).

**Table 2:** Background characteristics and skilled antenatal care services utilisation

Variable	Frequency = 190,903	Percentage (100%)	Proportion of women who utilized the services of skilled personnel during antenatal care
*Age*			
15 – 19	11,177	5.9	77.1
20 – 24	40,238	21.0	77.2
25 – 29	50,218	26.3	77.1
30 – 34	40,755	21.4	76.5
35 – 39	29,250	15.3	75.3
40 – 44	14,417	7.6	71.1
45 – 49	4,847	2.5	67.6
*Place of residence*			
Urban	60,584	31.8	90.0
Rural	130,319	68.2	69.5
*Level of education*			
No education	76,770	40.2	62.5
Primary	62,581	32.8	79.4
Secondary	44,575	23.3	91.3
Higher	6,977	3.7	97.3
*Wealth status*			
Poorest	41,105	21.5	64.9
Poorer	40,683	21.3	71.0
Middle	38,524	20.2	76.1
Richer	36,935	19.4	81.6
Richest	33,656	17.6	89.3
*Marital status*			
Married	148,131	77.6	74.5
Living with partner	36,121	18.9	80.0
Widowed	1,380	0.7	80.1
Divorced	1,224	0.6	87.4
Separated	4,047	2.2	89.4
*Partner’s educational level*			
No education	64,825	34.0	62.0
Primary	55,481	29.1	74.8
Secondary	56,448	29.5	88.7
Higher	14,149	7.4	94.4
*Frequency of listening to radio*			
Not at all	82,320	43.1	70.1
Less than once a week	38,388	20.1	77.6
At least once a week	65,304	34.2	81.7
Almost every day	4,891	2.5	86.5
*Frequency of watching television*			
Not at all	121,825	63.8	69.3
Less than once a week	23,4,6	12.3	81.6
At least once a week	37,229	19.5	90.0
Almost every day	8,443	4.4	95.8
*Getting medical help for self: getting permission to go*			
Big problem	36,541	19.1	70.1
Not a big problem	154,362	80.9	77.7
*Getting medical help for self: getting money needed for treatment*			
Big problem	104,924	55.0	71.3
Not a big problem	85,979	45.0	81.7
*Getting medical help for self: distance to health facility*			
Big problem	78,394	41.1	70.8
Not a big problem	112,509	58.9	79.6

### Predictors of skilled antenatal care services utilisation in sub-Saharan Africa

Table 3 shows results on the predictors of skilled ANC services utilisation in sub-Saharan Africa. In the adjusted model (Model 2), we found a likelihood of skilled ANC provision in urban areas compared to rural areas (aOR = 1.60, CI = 1.53–1.68). Women with a higher level of education were more likely to receive ANC from skilled providers compared to those with no formal education (aOR = 4.06, CI = 3.38–4.87). Women whose partners had higher education were also more likely to receive ANC from skilled providers compared to those whose partners had no formal education (aOR = 2.11, CI = 1.92–2.32). Richest women had higher odds of skilled ANC services compared to the poorest women (aOR = 3.16, CI = 2.91–3.42). The likelihood of receiving ANC from skilled providers was higher among women who listened to radio almost every day (aOR = 1.46, CI = 1.30–1.63) and those who watched television almost every day (aOR = 2.57, CI = 2.24–2.94) compared to those who never listened to radio nor watched television.

**Table 3:** Binary logistic regression results on the predictors of skilled antenatal care services utilisation among women in sub-Saharan Africa

Variable	Frequency (%)	Model 1 Crude Odds ratio (95% confidence interval)	Model 2 Adjusted Odds ratio (95% confidence interval)
*Age*			
15 – 19	11,177 (5.9)	0.99(0.95, 1.04)	0.97(0.91, 1.03)
20 – 24	40,238 (21.0)	1.01(0.98, 1.04)	0.99(0.95, 1.03)
25 – 29	50,218 (26.3)	Ref	Ref
30 – 34	40,755 (21.4)	0.97(0.94, 1.00)	1.04(0.99, 1.08)
35 – 39	29,250 (15.3)	0.92***(0.89, 0.95)	1.06*(1.02, 1.11)
40 – 44	14,417 (7.6)	0.75***(0.71, 0.78)	0.94(0.90, 1.00)
45 – 49	4,847 (2.5)	0.63***(0.59, 0.67)	0.86**(0.79, 0.93)
*Place of residence*			
Urban	60,584 (31.8)	3.35***(3.26, 3.45)	1.60***(1.53, 1.68)
Rural	130,319 (68.2)	Ref	Ref
*Level of education*			
No education	76,770 (40.2)	Ref	Ref
Primary	62,581 (32.8)	2.39***(2.33, 2.45)	1.56***(1.49, 1.63)
Secondary	44,575 (23.3)	5.15***(4.98, 5.33)	2.11***(1.99, 2.23)
Higher	6,977 (3.7)	19.31***(16.70, 22.33)	4.06***(3.38, 4.87)
*Wealth status*			
Poorest	41,105 (21.5)	Ref	Ref
Poorer	40,683 (21.3)	1.41***(1.37, 1.45)	1.43***(1.37, 1.49)
Middle	38,524 (20.2)	1.77***(1.71, 1.82)	1.70***(1.62, 1.77)
Richer	36,935 (19.4)	2.27***(2.20, 2.35)	2.13***(2.01, 2.25)
Richest	33,656 (17.6)	4.01***(3.86, 4.17)	3.16***(2.91, 3.42)
*Marital status*			
Married	148,131 (77.6)	Ref	Ref
Living with partner	36,121 (18.9)	1.32***(1.30, 1.37)	0.97(0.92, 1.02)
Widowed	1,380 (0.7)	1.46***(1.29, 1.65)	0.84*(0.72, 0.99)
Divorced	1,224 (0.6)	2.90***(2.49, 3.37)	0.89(0.75, 1.06)
Separated	4,047 (2.2)	2.66***(2.42, 2.93)	1.05(0.94, 1.18)
*Partner’s educational level*			
No education	64,825 (34.0)	Ref	Ref
Primary	55,481 (29.1)	1.90***(1.86, 1.95)	1.55***(1.48, 1.62)
Secondary	56,448 (29.5)	4.20***(4.08, 4.32)	1.76***(1.67, 1.85)
Higher	14,149 (7.4)	9.05***(8.43, 9.71)	2.11***(1.92, 2.32)
*Frequency of listening to radio*			
Not at all	82,320 (43.1)	Ref	Ref
Less than once a week	38,388 (20.1)	1.46***(1.42, 1.50)	1.32***(1.26, 1.38)
At least once a week	65,304 (34.2)	1.86***(1.81, 1.90)	1.29***(1.24, 1.35)
Almost every day	4,891 (2.5)	2.76***(2.56, 2.97)	1.46***(1.30, 1.63)
*Frequency of watching television*			
Not at all	121,825 (63.8)	Ref	Ref
Less than once a week	23,4,6 (12.3)	1.90***(1.84, 1.98)	1.21***(1.37, 1.49)
At least once a week	37,229 (19.5)	3.69***(3.56, 3.82)	1.40***(1.32, 1.48)
Almost every day	8,443 (4.4.)	8.48***(7.63, 9.43)	2.57***(2.24, 2.94)
*Getting medical help for self: getting permission to go*			
Big problem	36,541 (19.1)	0.66***(0.64, 0.68)	0.74***(0.71, 0.77)
Not a big problem	154,362 (80.9)	Ref	Ref
*Getting medical help for self: getting money needed for treatment*			
Big problem	104,924 (55.0)	Ref	Ref
Not a big problem	85,979 (45.0)	1.85***(1.81, 1.89)	0.96**(0.92, 0.99)
*Getting medical help for self: distance to health facility*			
Big problem	78,394 (41.1)	0.63***(0.61, 0.64)	0.75***(0.72, 0.77)
Not a big problem	112,509 (58.9)	Ref	Ref
*Survey country*			
Nigeria	20,320 (10.7)	Ref	Ref
Angola	5,674 (3.0)	1.85***(1.72, 1.98)	1.77***(1.62, 1.93)
Benin	7,957 (4.2)	2.37***(2.22, 2.52)	3.62***(3.37, 3.89)
Burundi	7,856 (4.1)	0.06***(0.05, 0.06)	0.05***(0.04, 0.05)
Burkina Faso	10,074 (5.4)	0.24***(0.23, 0.26)	0.34***(0.32, 0.36)
Cameroon	5,152 (2.7)	2.43***(2.24, 2.63)	2.67***(2.44, 2.91)
Chad	3,398 (1.8)	0.57***(0.53, 0.61)	1.08(0.99, 1.17)
Comoros	1,911 (1.0)	6.16***(5.18, 7.32)	7.93***(6.62, 9.50)
Congo DR	10,092 (5.3)	3.09***(2.90, 3.29)	3.62***(3.37, 3.89)
Congo	5,034 (2.6)	1.81***(1.69, 1.94)	1.72***(1.58, 1.89)
Cote d’lvoire	4,343 (2.2)	4.18***(3.79, 4.61)	7.44***(6.70, 8.25)
Ethiopia	7,070 (3.7)	0.62***(0.59, 0.66)	1.01(0.94, 1.08)
Gabon	2,545 (1.3)	5.05***(4.43, 5.75)	3.03***(2.60, 3.52)
Ghana	3,682 (1.9)	3.51***(3.18, 3.88)	3.62***(3.25, 4.04)
Gambia	4,893 (2.5)	76.33***(54.14, 107.63)	115.42***(81.72, 163.03)
Guinea	5,030 (2.6)	2.06***(1.91, 2.22)	4.05***(3.73, 4.40)
Kenya	6,196 (3.3)	8.68***(7.78, 9.68)	9.86***(8.78, 11.07)
Lesotho	2,251 (1.2)	10.59***(8.68, 12.92)	9.52***(7.75, 11.68)
Liberia	3,832 (2.0)	7.91***(6.97, 8.98)	12.16***(10.65, 13.90)
Malawi	11,050 (5.8)	10.06***(9.18, 11.03)	11.19***(10.15, 12.35)
Mali	6,130 (3.2)	1.62***(1.52, 1.73)	2.48***(2.30, 2.67)
Mozambique	6,988 (3.7)	6.13***(5.57, 6.74)	7.63***(6.89, 8.45)
Namibia	1,830 (1.0)	11.00***(8.87, 13.66)	8.80***(7.04, 11.01)
Niger	7,821 (4.1)	2.38****(2.22, 2.54)	4.30***(4.00, 4.63)
Rwanda	5,381 (2.8)	65.38***(48.02, 89.03)	63.59***(46.56, 86.84)
Sierra Leone	5,814 (3.1)	23.36***(19.55, 27.92)	49.04***(40.92, 58.78)
South Africa	616 (0.3)	11.79***(7.76, 17.92)	5.51***(3.59, 8.46)
Togo	4,525 (2.4)	0.37***(0.34, 0.39)	0.29***(0.26, 0.31)
Tanzania	5,684 (3.0)	0.05***(0.05, 0.06)	0.03***(0.02, 0.03)
Uganda	8,048 (4.2)	19.62***(17.05, 22.57)	20.02***(17.47, 23.40)
Zambia	5,313 (2.9)	15.05***(12.90, 17.55)	14.22***(12.12, 16.67)
Zimbabwe	4,166 (2.2)	8.39***(7.32, 9.61)	5.29***(4.59, 6.10)

*Ref* Reference category

^*^p < 0.05 **p < 0.01 ***p < 0.01

Lower odds of ANC from skilled providers was found among women aged 45–49 compared to those aged 25–29 (aOR = 0.86, CI = 0.79–0.93); widowed women compared to married women (aOR = 0.84, CI = 0.72–0.99); women who consider getting permission to visit the health facility as a big problem (aOR = 0.74, CI = 0.71, 0.77) compared to those who consider that as not a big problem; women who consider getting money needed for treatment as not a big problem compared to those who consider that as a big problem (aOR = 0.96, CI = 0.92–0.99); and women who consider distance to the health facility as a big problem compared to those who consider that as not a big problem (aOR = 0.75, CI = 0.72–0.77). The odds of ANC from skilled providers was found to be higher for women from Gambia (aOR = 115.45, CI = 81.72, 163.03) compared to women from Nigeria (see Table 3).

## Discussion

The present study sought to examine the prevalence and predictors of skilled ANC service utilisation in SSA. We found relatively high prevalence of skilled ANC utilisation across SSA countries included in this study. This prevalence is higher than the 49% prevalence of skilled ANC service utilisation that was reported by UNICEF [9]. Probably, the high prevalence of skilled ANC utilisation across SSA may be due to increasing rate of urbanisation in SSA which tends to bring women closer to healthcare facilities where they can have access to skilled personnel [14]. Also, the findings may be due to overall increase in education and improvement in socio-economic status of the populace. However, there were between country differences. While Burundi reported the lowest prevalence of skilled ANC service utilisation, Rwanda and the Gambia had the highest prevalence of skilled ANC service utilisation. Protracted conflicts and instability may explain why women in Burundi had low prevalence and lower odds of having ANC by a skilled provider. As stated in the works of Chi et al. [15] armed conflict has been described as an important contributor to the social determinants of health and a driver of health inequity, including maternal health. These conflicts can severely reduce access to maternal health services and thus lead to poor maternal health outcomes for a period beyond the conflict itself. In the case of Rwanda, the high prevalence and odds of women to utilise ANC services may be due to ripple effects from the implementation of comprehensive health sector reforms after the 1994 genocide which brought about substantial improvement in maternal healthcare dimensions including skilled ANC service utilisation [16].

Concerning the predictors of skilled ANC service utilisation, our study reveals that urban dwelling women were more likely to receive ANC from skilled personnel as compared to women in rural residences. Analogous findings have been reported in Ghana [5, 14]. Usually, there are rural–urban disparities in the allocation of healthcare resources, with rural residences being disproportionately disadvantaged [5]. As such, it is very easy for urban dwelling women to have access to ANC services from a skilled provider as compared to those in rural areas. Also, in most cases, skilled providers refuse postings to rural communities; rather, they prefer to be centralised in urban areas where they can have access to various social amenities and socio-economic prospects [17, 18]. Thus, limiting the likelihood of rural dwelling women to receive ANC services from skilled providers. Another possible explanation for observed rural–urban differences could be the long distance that women in rural areas have to cover in order to get access to skilled ANC services.

Women with a higher level of education were four times more likely to utilise ANC services from skilled providers compared to those with no formal education. This result is congruent with findings from previous studies [5, 19]. Formal education has the tendency to empower women and raise their awareness about the need to receive care from skilled providers as well as the dangers of receiving ANC from unskilled providers. Relatedly, women whose partners had secondary or higher education were more likely to utilise ANC services from skilled providers, compared to those whose partners had no formal education. A likely explanation is that, partners with higher educational attainment have the tendency to encourage their partners to utilise ANC services from skilled providers.

Our study also indicates that compared to women in the poorest wealth index, those in the richest wealth index were more likely to utilise ANC services from skilled providers. The finding is consistent with earlier studies by Ganle et al. [20] and Atunah-Jay et al. [21]. Higher wealth index provides women with autonomy and control over resources. Hence, empowering women to take decisions to utilise skilled ANC services [22]. This affirms our findings that women who consider getting money needed for treatment as not a big problem have higher likelihood of utilising ANC services from skilled providers as compared to those who consider that as a big problem. Thus, it is imperative to improve the economic status of women in order to reduce the effects of out-of-pocket-payment and improve utilisation of skilled ANC services.

Compared to those aged 20–24 years, women aged 45–49 were less likely to utilise ANC services from skilled providers. Thus, suggesting that older reproductive age is associated with lower odds of skilled ANC service utilisation. This finding is inconsistent with earlier studies from Nepal [23] and Kenya [24]. Possibly, older women in their reproductive age believe they have amassed a wealth of experience from their previous births. As such, utilising ANC from skilled providers no longer becomes a priority to them. Our study also reveals that exposure to media (listening to radio and watching TV) significantly predicted the likelihood of utilising skilled ANC services. The present finding is corroborated by the findings of Girmaye and Berhan [25]. Exposure to media provides women the opportunity to have access to information about the importance of utilising skilled ANC services. This may explain why those who were exposed to media were more likely to utilise ANC services from skilled providers.

### Policy implications

The findings from this study have some implications on policy and planning. Our findings underscore the need to leverage on the media to reach out to women with health information and messages about the benefits of utilising skilled ANC services. Skilled ANC service provision in rural areas would have to be prioritised. Governments across SSA must endeavour to establish community-based healthcare facilities in rural areas like the Community-based Health Planning and Services (CHPS) initiative in Ghana in order to reduce the effect of distance to healthcare facility on the likelihood of utilising skilled ANC services.

### Strengths and weaknesses

Given that we relied on the DHS which uses cross-sectional design, we are unable to establish causality for the predictors of skilled ANC service utilisation. Also, we were limited to variables that are available in the dataset. As such, we were unable to explore how important factors such as cultural norms and patriarchal ideologies predict skilled ANC utilisation. The data was self-reported; hence, there is the possibility of recall and social desirability bias. Some of our findings like the rural–urban differences in skilled ANC service utilisation may be influenced by the number of ANC services available in both rural and urban areas. Yet, the use of secondary data does not allow us to test this hypothesis as it does not have the data necessary to support this. Also, because we pooled data in our analysis, there is the possibility of overestimation of the prevalence of skilled ANC service utilisation. Our study also did not look at the spatial analysis. Nevertheless, we acknowledge that the use of a nationally representative dataset allows us to generalise the study findings to the countries considered in our study. Also, we cautiously claim that our study is the first of its kind to investigate the predictors of skilled ANC utilisation from a multi-country or regional perspective, which is a great contribution to existing literature.

## Conclusion

Recognising the importance of ANC service provision by skilled providers, we examined the predictors skilled ANC utilisation in SSA. We conclude that SSA has high prevalence of skilled ANC service utilisation, however, there are substantial country differences that need to be prioritised. Also mother’s educational attainment, partners’ educational attainment, urban residency, higher wealth status and exposure to media were facilitating predictors of skilled ANC utilisation. Old age, being widowed and far distance to health facility were factors that predicted lower likelihood of skilled ANC utilisation. There is, therefore, the need to intensify female formal education, invest in community-based healthcare facilities in rural areas and leverage on the media in advocating for skilled ANC service utilisation.

## Data Availability

The dataset is freely available for download at https://dhsprogram.com/data/available-datasets.cfm.

## Abbreviations

ANC: Antenatal care
CHPS: Community-based Health Planning and Services
SSA: Sub-Saharan Africa

## References

[1] World Health Organization. Trends in maternal mortality: 1990 to 2013: estimates by WHO, UNICEF, UNFPA, The World Bank and the United Nations Population Division: executive summary. (No. WHO/RHR/14.13). World Health Organization; 2014. https://apps.who.int/iris/handle/10665/112697.

[2] World Health Organization. Maternal mortality: key facts. 2019. [Internet] Available: https://www.who.int/news-room/fact-sheets/detail/maternal-mortality. Accessed 2 May 2022.

[3] Bongaarts J. WHO, UNICEF, UNFPA, World Bank Group, and United Nations Population Division Trends in Maternal Mortality: 1990 to 2015 Geneva: World Health Organization; 2015. p. 726.

[4] World Health Organization. Maternal Mortality. 2016. [Internet]. Available from: http://www.who.int/mediacentre/factsheets/fs348/en/. Accessed 2 May 2022

[5] Dickson KS, Darteh EK, Kumi-Kyereme A, Ahinkorah BO (2018). Determinants of choice of skilled antenatal care service providers in Ghana: analysis of demographic and health survey. Matern Health Neonatol Perinatol.

[6] Begum N, Rahman M, Rahman MM, Nayan SK, Zinia SN, Khan SZ (2014). Utilization of antenatal care services in a selected rural area in Bangladesh. North Int Med Coll J.

[7] Tunçalp Ӧ, Pena-Rosas JP, Lawrie T (2017). WHO recommendations on antenatal care for a positive pregnancy experience-going beyond survival. BJOG.

[8] Ali N, Elbarazi I, Alabboud S, Al-Maskari F, Loney T, Ahmed LA. Antenatal care initiation among pregnant women in the United Arab Emirates: the Mutaba’ah study. Front Public Health. 2020;11(8):211. 10.3389/fpubh.2020.00211 PMC7300181 32596198

[9] UNICEF (2016). Global databases, based on MICS, DHS and other nationally representative sources, 2008–2014.

[10] Lakew S, Ankala A, Jemal F (2018). Determinants of client satisfaction to skilled antenatal care services at Southwest of Ethiopia: a cross-sectional facility based survey. BMC Pregnancy Childbirth.

[11] Tweheyo R, Konde-Lule J, Tumwesigye NM, Sekandi JN (2010). Male partner attendance of skilled antenatal care in peri-urban Gulu district Northern Uganda. BMC Pregnancy Childbirth.

[12] Aliaga A, Ruilin R (2006). Cluster optimal sample size for demographic and health surveys. In 7th International Conference on Teaching Statistics–ICOTS.

[13] Von Elm E, Altman DG, Egger M, Pocock SJ, Gøtzsche PC, Vandenbroucke JP (2007). The Strengthening the Reporting of Observational Studies in Epidemiology (STROBE) statement: guidelines for reporting observational studies. Bull World Health Organ.

[14] Dickson KS, Darteh EK, Kumi-Kyereme A (2017). Providers of antenatal care services in Ghana: evidence from Ghana demographic and health surveys 1988–2014. BMC Health Serv Res.

[15] Chi PC, Bulage P, Urdal H, Sundby J (2015). A qualitative study exploring the determinants of maternal health service uptake in post-conflict Burundi and Northern Uganda. BMC Pregnancy Childbirth.

[16] Bucagu M, Kagubare JM, Basinga P, Ngabo F, Timmons BK, Lee AC (2012). Impact of health systems strengthening on coverage of maternal health services in Rwanda, 2000–2010: a systematic review. Reprod Health Matters.

[17] Nwankwo ON, Ugwu CI, Nwankwo GI, Akpoke MA, Anyigor C, Obi-Nwankwo U, Andrew S, Nwogu K, Spicer N (2022). A qualitative inquiry of rural-urban inequalities in the distribution and retention of healthcare workers in southern Nigeria. PLoS ONE.

[18] Adzei FA, Atinga RA. Motivation and retention of health workers in Ghana's district hospitals: addressing the critical issues. J Health Organ Manag. 2012;26(4-5):467–85. 10.1108/14777261211251535. 10.1108/14777261211251535 23115900

[19] Le Meur N, Gao F, Bayat S (2015). Mining care trajectories using health administrative information systems: the use of state sequence analysis to assess disparities in prenatal care consumption. BMC Health Serv Res.

[20] Ganle JK, Parker M, Fitzpatrick R, Otupiri E (2014). Inequities in accessibility to and utilisation of maternal health services in Ghana after user-fee exemption: a descriptive study. Int J Equity Health.

[21] Atunah-Jay SJ, Pettingell S, Ohene SA, Michael Oakes J, Borowsky IW (2013). The relationship between antenatal provider type and maternal care in rural Ghana: a cross-sectional study. Tropical Med Int Health.

[22] Aboagye RG, Okyere J, Ahinkorah BO, Seidu AA, Zegeye B, Amu H, Yaya S (2022). Health insurance coverage and timely antenatal care attendance in sub-Saharan Africa. BMC Health Serv Res.

[23] Joshi C, Torvaldsen S, Hodgson R, Hayen A (2014). Factors associated with the use and quality of antenatal care in Nepal: a population-based study using the demographic and health survey data. BMC Pregnancy Childbirth.

[24] Ochako R, Gichuhi W (2016). Pregnancy wantedness, frequency and timing of antenatal care visit among women of childbearing age in Kenya. Reprod Health.

[25] Girmaye M, Berhan Y (2016). Skilled antenatal care service utilization and its association with the characteristics of women’s health development team in Yeky District, south-west Ethiopia: a multilevel analysis. Ethiop J Health Sci.

